# The Tao of Hematopoietic Stem Cells: Toward a Unified Theory of Tissue Regeneration

**DOI:** 10.1100/tsw.2002.162

**Published:** 2002-04-10

**Authors:** Kevin D. Bunting, Robert G. Hawley

**Affiliations:** Hematopoiesis Department, Cell Therapy Research and Development, American Red Cross Holland Laboratory, 15601 Crabbs Branch Way, Rockville, MD 20855, USA

**Keywords:** hematopoietic stem cells, stem cell plasticity, transdifferentiation, tissue regeneration, cell therapy

## Abstract

Hematopoietic stem cells (HSCs) are the best studied of the tissue-specific stem cells. By definition, HSCs have long been regarded as restricted to formation of blood cells of both the lymphoid and myeloid lineages. HSCs residing in the bone marrow microenvironment have self-renewal capacity and can repopulate the hematopoietic system of irradiated transplant recipients for the lifetime of the individual. Therefore, HSCs are extremely important targets for gene therapy applications aimed toward the treatment of inherited and acquired blood disorders. However, recent studies have suggested that a subpopulation of HSCs may have the ability to contribute to diverse cell types such as hepatocytes, myocytes, and neuronal cells, especially following induced tissue damage. Preclinical amelioration of liver disease and myocardial infarcts by HSC-enriched bone marrow cell populations raises the possibility that HSC transplants have the potential to provide therapeutic benefit for a wide variety of diseases. These surprising findings contradict the dogma that adult stem cells are developmentally restricted. Extrapolation of these findings to the clinic will be facilitated by prospective identification of the stem cells that possess this developmental plasticity. Furthermore, characterization of the signaling pathways and molecular determinants regulating the remarkable transdifferentiation capacity of these stem cells may provide insight into novel approaches for modulating frequency of differentiative potential.

## HEMATOPOIETIC STEM CELLS AND CLASSICAL BLOOD-FORMING ACTIVITY

Hematopoietic stem cells (HSCs) are characterized by their capacity to self-renew and their ability to support hematopoiesis for the lifetime of an individual. These properties make stem cells much more valuable from a therapeutic standpoint than the progenitor population, which has limited self-renewal potential and thus cannot provide long-term reconstitution of the hematopoietic system. Studies of HSC biology have been most extensively conducted in mice. This is because, with the exception of clinical gene transfer trials, an analogous procedure is not available for investigational studies in humans, while transplant studies with nonhuman primates are very costly. In the mouse model, transplantation of total bone marrow cells has been shown to fully repopulate both the lymphoid and myeloid lineages of lethally irradiated recipients[1]. As described below, the stem cell transplant assay has facilitated efforts to prospectively identify and quantify HSCs. It has also been used to optimize gene delivery protocols to investigate effects on HSC function and for preclinical animal modeling of HSC gene therapy applications[2,3].

A major advance in the field of HSC biology was the extensive characterization of the phenotype of murine HSCs. This work revealed that adult bone marrow–derived HSCs express the c-Kit tyrosine kinase receptor and the stem cell antigen Sca-1. Selection for cells not expressing markers of lineage differentiation further defined the HSC population as having a c-Kit$^{+}$Thy-1.1$^{low}$Lin$^{neg}$Sca-1$^{+}$ phenotype[4,5,6,7]. Murine HSCs variably express the sialomucin CD34, depending on developmental stage and cell cycle status[8,9,10,11]. Fluorescent dye staining approaches have also been used to define HSCs functionally based on efflux capacity and staining profile. Rhodamine 123 (Rho123) staining of bone marrow cells was used to demonstrate that high efflux of Rho123 was associated with the most primitive HSCs, whereas cells that did not efflux Rho123 well and stained more brightly could only provide short-term repopulation[12,13,14]. Hoechst 33342 is another fluorescent dye used for isolation of HSC fractions[15,16]. The combination of both Rho123 and Hoechst 33342 staining has demonstrated that Hoechst$^{low}$Rho$^{low}$ cells are highly enriched for HSC activity[17,18]. A dual-wavelength flow cytometric analysis based on differential Hoechst 33342 staining was recently developed that identifies an HSC fraction termed *side-population* (SP) cells, which have been shown to give rise to all mature blood lineages in transplanted mice[16].

Several xenogeneic transplant assays have been developed for human hematopoietic repopulating cells. These generally involve transplanting candidate human HSCs into severe combined immunodeficient (SCID) mice, SCID mice crossed with nonobese diabetic (NOD) mice deficient in macrophage function and having inherently low natural killer cell activity (NOD/SCID mice), or beige/nude/xid mice, occasionally followed by treatment with recombinant human cytokines or engineered stromal cell lines expressing human cytokines[19,20,21]. The human SCID-repopulating cells give rise to high levels of committed and multilineage human myeloerythroid progenitors as well as mature B lymphoid and myeloid cells in the murine bone marrow for at least 4 months. Analogous results have been obtained using other xenograft models, such as the SCID-human bone system in which a human fetal bone fragment is implanted in SCID mice as a supportive human hematopoietic microenvironment[22] and the preimmune fetal sheep transplant assay[23].

To date, clinical protocols involving enrichment for human HSCs generally utilize cells expressing CD34[24,25]. Further isolation and characterization of specific CD34$^{+}$ subsets have defined more primitive precursors with hematopoietic repopulating activity as being CD34$^{+}$ and lacking lineage differentiation markers (CD34$^{+}$Lin$^{neg}$). Using NOD/SCID and sheep xenograft assays, it was found that the *in vivo* marrow engrafting ability of CD34$^{+}$Lin$^{neg}$ cells mainly resides in a subset of cells lacking the CD38 surface molecule[26,27]. Additional studies have indicated that very rare CD34$^{neg}$ engrafting cells exist and that human hematopoietic repopulating activity correlates with expression of the CD133 (formerly AC133) surface antigen[28,29,30,31]. Like mouse HSCs, human HSCs are positive for Thy-1 expression; they also express c-Kit as well as a surface marker, CD59, related to Sca-1[32,33,34]. Differential efflux of Hoechst 33342 and Rho123 also enriches for primitive human hematopoietic precursors, with the SP fraction containing candidate HSCs[18,35,36,37].

## BONE MARROW CONTAINS OTHER MULTIPOTENT STEM CELLS

A second type of stem cell can be found within the bone marrow microenvironment that lacks blood-forming ability but can differentiate into multiple mesodermally derived tissues[38]. Termed *mesenchymal stem cells* (MSCs), these multipotent adult stem cells can give rise to bone marrow stroma, bone, cartilage, tendon, fat, and, at least in rodents, skeletal muscle[39,40,41]. Very recently, it has been claimed that human bone marrow contains a third type of stem cell, designated *mesodermal progenitor cells* (MPCs), possessing an even broader range of differentiation potential than MSCs[42]. MPCs can apparently differentiate not only into all MSC-derived cell types (including skeletal muscle–like cells) but also into endothelial cells. The *in vivo* hematopoietic repopulating ability of MPCs was not examined. However, MPCs could not be induced to differentiate into hematopoietic cells *in vitro* under the conditions employed. Unpublished results referred to by the investigators suggest that MPCs may be capable of differentiating into nonmesodermal cell types, such as neuroectoderm. Therefore, until definitive studies are carried out, it remains a formal possibility that in some cases bone marrow–derived cells with the capacity to regenerate extrahematopoietic tissues might be MPCs or, in certain instances, MSCs.

## PROGRESS IN IDENTIFYING STEM CELLS FROM SKELETAL MUSCLE AND THE BRAIN

Like the hematopoietic system, other organs (skeletal muscle, intestine, skin) in the adult have stem cells that persist throughout life and contribute to regeneration. However, characterization of the stem cell phenotype is much less well defined in these systems. Similarly, the capacity of stem cells to regenerate other tissues (liver, heart, brain) following injury is not well documented. This paucity of information has been due in large part to the lack of a stem cell assay comparable to the transplantation of bone marrow cells for hematopoietic reconstitution.

It is known that normally highly quiescent skeletal muscle tissue can be activated and regenerate in response to weight-bearing stress or trauma[43]. Mononuclear myogenic progenitors, called *satellite cells*, can be found between the sarcolemma and the basal lamina of the muscle fiber. Upon activation, satellite cells give rise to myoblasts and begin expressing myogenic regulatory factors such as Myf5 and MyoD, generating daughter precursor cells that undergo multiple rounds of cell division before terminally differentiating and fusing with new or existing myofibers[44]. Because the total number of satellite cells in adult muscle remains constant over repeated cycles of regeneration, satellite cells have been suggested to form a population of monopotential muscle stem cells. Recent studies have identified SP cells in adult mouse skeletal muscle that can also participate in myogenic regeneration[45]. The muscle SP cells isolated, which were c-Kit$^{neg}$Sca-1$^{+}$Lin$^{neg}$, were suggested to give rise to satellite cells. Regeneration of mouse skeletal muscle has also been demonstrated by two groups with muscle-derived myogenic cells isolated using a preplate technique and direct selection for Sca-1$^{+}$ cells[46,47]. Examination of CD34 expression yielded differing results and the relationship amongst the different Sca-1$^{+}$ muscle cell precursor populations remains to be determined. However, it is clear that muscle SP and satellite cells represent distinct cell populations, as evidenced by the complete absence of the latter but not the former in the muscles of Pax7 knockout mice[48]. On the basis of these results, it has been proposed that upregulation of Pax7 in muscle-derived multipotent stem cells, perhaps SP cells, induces satellite cell specification. This concerted temporal expression profile of transcription factors during skeletal muscle development suggests that ectopic triggering of myogenic signaling pathways may permit directed muscle regeneration in future stem cell therapies. In contrast to skeletal muscle, the ability of cardiac muscle to repair itself following myocardial infarction has not been demonstrated despite recent positive findings that cardiomyocytes can be generated from bone marrow stromal cells[49] *in vitro* and a report that actively cycling cells can be detected in the peri-infarct region in human patients[50].

As with cardiac muscle regeneration, neuronal regeneration has generally been believed not to occur following damage due to ischemia or other blunt trauma. However, demonstration of cell cycling in the brain of rhesus macaques was recently reported, suggesting that a normal physiological turnover of brain cells might occur *in vivo*[51]. During embryonic development, the neural crest of the fetal peripheral nerve has been shown to be an area enriched for neural stem cells (NSCs)[52]. In the adult, NSCs have been identified in a variety of anatomical locations within the brain[53,54,55,56,57], increasing the complexity of this field. Clonal lineage tracing studies raise the possibility that these disparate stem cell pools may be descendants of a common NSC[58]. Neural precursors cultured from the ventricular and subventricular zone form colonies called *neurospheres.* From 3 to 4% of the cells within the neurospheres are considered to be NSCs in that they expand *in vitro* and retain the capacity to differentiate into the three primary brain cell types — neurons, astrocytes, and oligodendrocytes. Until recently, this has been the method for defining multipotent NSCs; but recent work in the field of neural stem/progenitor cell biology has made significant progress in demonstrating that mouse NSCs can be prospectively defined based on expression of nestin plus low levels of expression of peanut agglutinin-binding glycoproteins and heat-stable antigen (CD24)[57,59]. Parallels with the hematopoietic system are also emerging[60]. Neurosphere cultures have been shown to contain SP cells[61], and candidate human NSCs characterized as neurosphere-initiating cells capable of differentiating into neurons and glial cells express the human HSC marker CD133[62]. Moreover, screening for HSC-specific genes expressed in neurospheres revealed a number of candidate genes that could play regulatory roles in both neuropoiesis and hematopoiesis[63,64]. Unlike HSCs, NSCs can be propagated *in vitro,* which is a potential advantage for future clinical use of these cells. Furthermore, the ability to transplant NSCs *in vivo,* which in some instances form morphologically normal brain cells and electrically active synapses, has opened the door to possible stem cell–based therapies for a range of neurological disorders that include Alzheimer's and Parkinson's diseases[65,66,67].

## UNEXPECTED DIFFERENTIATIVE POTENTIAL OF HSCS AND OTHER TISSUE-SPECIFIC STEM CELLS

Transplantation studies in rodents with unfractionated bone marrow first raised the possibility that HSCs might possess differentiative potential outside of the hematopoietic system. Thus, bone marrow cells have been observed to contribute to skeletal muscle[68], smooth muscle[69], astroglial and neuronal cells[70,71,72], and hepatic oval cells[73]. Examination of archival autopsy and biopsy liver specimens of bone marrow transplant patients has provided similar evidence for transdifferentiation of circulating stem cells into hepatocytes in humans[74,75].

Mulligan and colleagues transplanted bone marrow SP cells into the *mdx* mouse, a model for Duchenne muscular dystrophy, and demonstrated restoration of dystrophin expression in up to 4% of myofibers, with 10 to 30% of these containing fused donor nuclei[45]. In reciprocal experiments, these investigators isolated muscle SP cells and showed that intravenous injection of 7,000 to 20,000 cells had the ability to reconstitute the hematopoietic compartment of irradiated *mdx* mice, with engraftment levels ranging from 30 to 91%[45]. The muscle SP cells were also capable of contributing at low levels to skeletal muscle engraftment. Several other groups have confirmed that cells derived from murine skeletal muscle have hematopoietic repopulating potential following intravenous transplantation[76,77,78]. Goodell and colleagues transplanted muscle cells that had been cultured for 5 days and, using a competitive repopulation assay, showed that these cells had approximately 10 to 14 times more hematopoietic repopulating activity than whole bone marrow[76]. These studies have generated much excitement in the stem cell biology field because of the possibility that human HSCs might one day be useful for treating muscular dystrophy and other disorders of nonhematopoietic tissues by systemic cell delivery. Conversely, if it were possible to maintain and expand human muscle–derived stem cells with hematopoietic repopulating potential *in vitro,* muscle biopsies could provide an alternative source of HSCs for clinical transplantation. From a developmental perspective, muscle tissue, like bone marrow and blood, is of mesodermal origin. The question is raised therefore as to whether HSCs or muscle stem cells are capable of transdifferentiating into the other mesodermal derivative or whether the cells under study are more primitive stem cells with both hematopoietic and myogenic activity. Hoechst 33342 dye efflux studies have indicated that murine bone marrow SP cells are predominantly c-Kit$^{+}$Sca-1$^{+}$CD45$^{+}$, whereas the muscle SP cells that were transplanted into the *mdx* mice were mainly c-Kit$^{neg}$Sca-1$^{+}$CD45$^{neg}$[16,35,45], implying that reciprocal transdifferentiation of tissue-specific stem cells has occurred. As mentioned above, myogenic satellite cells are absent in Pax7 knockout mice; nevertheless the muscles of Pax7 knockout mice contain SP cells that are capable of hematopoietic differentiation[48]. This result could be interpreted to mean that the myogenic and hematopoietic precursors in muscle are distinct. Consistent with this notion, Kawada and Ogawa reported that hematopoietic repopulating cells in muscle originated from transplanted bone marrow[78]. On the other hand, in a separate set of experiments performed by Bresolin and colleagues, Sca-1$^{+}$CD34$^{+}$ muscle-derived stem cells were isolated that exhibited myogenic and myeloid differentiation potential *in vitro,* and could partially correct dystrophin deficiency in the *mdx* mouse[46]. More stringent prospective isolation of these various bone marrow and muscle stem cell populations and clonality studies are necessary to elucidate their identities and reveal their full developmental potential. Irrespective of the outcomes, it is envisioned that much will be learned that will provide the framework for future clinical application.

As noted above, comparisons have been drawn between hematopoietic and nervous system development[60,63]. Preliminary accounts have indicated that intravenously injected murine bone marrow–derived cells give rise to macroglia and cells bearing neuronal antigens in the host brain[70,71,72]. Rat and human bone marrow stromal cells can be induced to express neuronal markers in culture[79] and murine bone marrow stromal cells transplanted into neonatal mouse brain can differentiate into astrocytes and neurofilament-containing cells[80]. Whether HSCs can adopt neural cell fates is not yet known. However, the converse appears to be true. Extraordinary plasticity of rodent NSCs and neural precursors has been described, including the adoption of hematopoietic cell fate. Thus, rat oligodendrocyte precursors can be reprogrammed to become NSCs by culturing *in vitro* in the presence of basic fibroblast growth factor[81] while mouse NSCs have been shown to transdifferentiate into hematopoietic cells *in vivo*[82] and into skeletal muscle *in vitro* and *in vivo*[57,83]; they have also been shown to contribute to cells representative of all three germ layers in chimeric chick and mouse embryos[84]. Notably, cultured human NSCs also appear to possess hematopoietic potential[85] as well as myogenic potential[83], raising the possibility that they will also exhibit a broad developmental repertoire that might eventually be exploited for the treatment of a wide variety of diseases.

Many of the bone marrow plasticity studies have been criticized for their lack of well-characterized donor populations and clonal analyses. Toward these goals, Krause and colleagues reported that CD34$^{+}$Lin$^{neg}$ bone marrow cells were capable of hepatic engraftment[86]. Using limiting dilution to transplant an enriched HSC population obtained via elutriation and lineage depletion, these investigators subsequently demonstrated that individual cells with bone marrow–homing ability and increased expression of CD34 and Sca-1 were capable of differentiating into hematopoietic cells as well as epithelial cells of the liver, lung, gastrointestinal tract, and skin in secondary recipients[87]. Other elegant studies by Lagasse and colleagues in an animal model of tyrosinemia type I, the FAH$^{-/-}$ mouse, have conclusively documented that HSCs are the cells that give rise to hepatocytes. As few as 50 highly purified c-Kit$^{+}$Thy-1.1$^{low}$Lin$^{neg}$Sca-1$^{+}$ HSCs injected intravenously were shown to reconstitute hematopoiesis and restore liver function[88].

Cell-based therapies for treating heart disease have been gaining interest over the past several years. Tomita and colleagues first demonstrated the potential utility of autologous bone marrow transplantation as a therapeutic approach to limit the progress of cardiac damage following myocardial infarction[89]. Ensuing compelling work by Orlic and colleagues showed that murine bone marrow cells enriched for c-Kit$^{+}$Lin$^{neg}$ HSCs can regenerate heart tissue following induced myocardial infarction, improving cardiac hemodynamics and ameliorating disease outcome[90]. In other recent studies, Goodell and colleagues reported that murine bone marrow SP cells with a c-Kit$^{+}$Sca-1$^{+}$ phenotype can engraft ischemic heart tissue at a low frequency (0.02%)[91]. Donor-derived endothelial cells were also identified, primarily in small blood vessels adjacent to the infarct (3.3%). Bone marrow–derived MSCs and circulating endothelial cell progenitors had previously been postulated to contribute new vessels to the postischemic myocardium[92,93,94,95]. These new findings provide support for the concept that HSCs within the bone marrow are capable of developing into cardiomyocytes and endothelial cells after heart damage. Collectively, the above reports argue that under certain conditions murine HSC subpopulations may possess a remarkable ability to cross lineage boundaries and give rise to cells representative of mesodermal, endodermal, and ectodermal origin (Fig. 1).

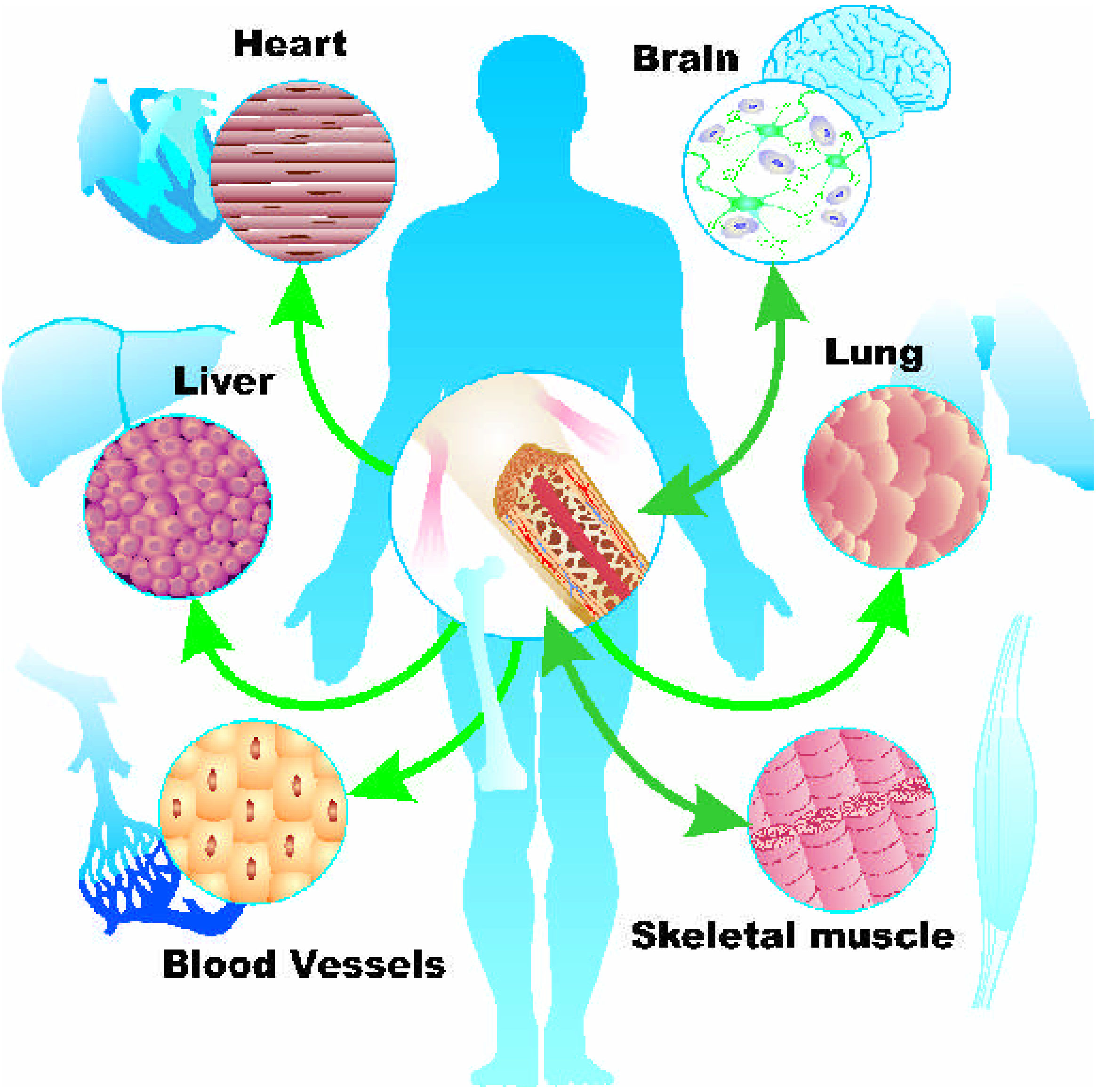

**FIGURE 1.** Transdifferentiation potential of bone marrow–derived HSCs and hematopoietic capacity of brain and skeletal muscle stem/precursor cells as inferred from animal models and examination of tissue specimens of bone marrow transplant patients. See text for details. (After the cover illustration of Hematopoietic Stem Cells 2000: Basic and Clinical Sciences, *Ann. N.Y. Acad. Sci.* **938,** 2001.)

## CHARACTERIZATION OFTHE GENETIC PROGRAMS RESPONSIBLE FOR STEM CELL PHENOTYPE, PHYSIOLOGIC FUNCTION, AND TRANSDIFFERENTIATION POTENTIAL

The genetic programs involved in stem cell self-renewal and differentiation remain largely unknown, although data is accumulating that implicates the Wnt, Hedgehog, and Notch signaling pathways in stem cell regulation[96]. Progress in applying global gene expression analysis technology, such as the Stem Cell Database (SCDb; http://stemcell.princeton.edu/) created by Lemischka and Overton, will be required to advance this field[97]. The SCDb catalogs genes that are differentially expressed in murine fetal liver HSCs relative to differentiated cell types. Future development of comparable databases and microarray representations of other stem cells will provide a better understanding of the extremely complex orchestration of gene regulation involved. If there is substantial overlap of genetic programs across species[98,99] and amongst different stem cell populations[63,64], a stem cell “genetic blueprint” may ultimately be delineated. One common transcript of note, which has already been identified in both HSCs and neural precursors, encodes the ATP-binding cassette (ABC) transporter family member called *breast cancer resistance protein 1* (BCRP1; also known as ABCG2)[63,64]. Expression of BCRP1 has recently been reported in SP cells from diverse sources, including murine bone marrow and skeletal muscle, as well as rhesus monkey bone marrow and murine embryonic stem cells[100]. Moreover, as previously demonstrated for another ABC transporter family member, the multidrug resistance 1 gene[101,102], enforced expression of BCRP1 in murine bone marrow cells caused an expansion of the SP phenotype[100]. BCRP1 expression may thus prove to be a universal molecular determinant of stem cell function. A role for ABC transporter efflux activity in stem cells suggests that removal of key differentiation-promoting substrates might be a central mechanism for maintenance of the stem cell state[103].

## IMPORTANT QUESTIONS REMAINING TO BE ADDRESSED

A common theme throughout the plasticity studies conducted to date has been the requirement for some level of injury (e.g., gamma irradiation, liver disease, heart damage) to stimulate regenerative signals that recruit the transplanted cells. Egress from the bone marrow to the site of injury appears to be a factor limiting normal physiologic tissue repair by HSCs, since mobilization with granulocyte-colony-stimulating factor and stem cell factor dramatically increased regeneration of cardiomyocytes in a myocardial infarct model of ischemic heart disease[104]. Elucidation of the mechanisms operating may facilitate the development of novel noninvasive stem cell therapies. The common expression of c-Kit and/or Sca-1 and the SP phenotype between stem cells of different murine tissues suggests that populations enriched for these markers might include putative adult pluripotent stem cells. Additional markers are needed so that the relevant stem cell populations can be prospectively identified and tagged for *in vivo* tracking[105,106,107,108]. The degree of restoration of function is a central issue that has obvious ramifications for the future success of stem cell–based therapeutics. Ideally, correction of a physiological defect, initially in an animal model, should be the yardstick by which the extent of functional tissue regeneration is measured. The durability of tissue regeneration from transdifferentiated stem cells will need to be ascertained since, by definition, stem cells should be capable of restoring the tissue for the lifetime of the individual. If the signaling mechanisms required for stem cell transdifferentiation can be defined, then the door might be opened to biochemical or genetic manipulation of cell fate decisions. For example, conditional ectopic expression of Pax7 in human HSCs might efficiently direct muscle-specific differentiation for ultimate treatment of muscular dystrophy[48]. If these goals can be achieved, the possibility exists that even in the absence of adult pluripotent stem cells comparable to embryonic stem cells, stem cell plasticity of HSCs and other tissue-specific stem cells might be exploited to restore tissue following injury. It is important to emphasize the fact that *ex vivo* expansion of human HSCs has been sought after for many years but has not yet been achieved[109]. Therefore, further understanding of the mechanisms — such as those involving ABC transporters — that might increase the number of HSCs in culture is required. Alternatively, neural (and perhaps muscle) stem cells, which can be readily expanded *in vitro*, might prove to be surrogate HSC sources for certain clinical transplant indications. Still, others would contend that embryonic stem cells hold the greatest potential for cell replacement therapies[110,111]. However, there are ethical issues surrounding the derivation and use of human embryonic stem cells. Only through comparisons of the basic biology of all stem cell types will the extent to which they might prove useful in a particular therapeutic context become apparent. (For an in-depth review of research involving stem cells from adult, embryonic, and fetal tissue sources, go to http://www.nih.gov/news/stemcell/scireport.htm.) In support of such studies, the National Stem Cell Resource has recently been established at the American Type Culture Collection (http://pasteur.atcc.org/StemCell/index.html)[112].

## ACKNOWLEDGMENTS

This work was supported in part by National Institutes of Health grants HL65519 and HL66305 (to R.G.H.).

## NOTE ADDED IN PROOF

Two recent papers have corroborated the results of Kawada and Ogawa showing that itinerant bone marrow-derived cells in muscle, not transdifferentiated muscle stem cells, are responsible for the hematopoietic repopulating activity detected in this tissue[78,113,114]. Since heterogeneity of stem cell types within a particular tissue is also a plausible explanation for some of the other plasticity results that have been reported[115], these new findings highlight the importance for prospective identification of somatic stem cell populations and clonal lineage analysis to document claims of nonconventional cell fate transitions. In a related development, Iscove, van der Kooy, and their colleagues recently described their failure in repeated efforts to reproduce studies that demonstrated hematopoietic differentiative potential of transplanted NSCs[82,116]. Instead, the group noted marked changes in the growth properties of the NSCs during extended passaging in culture; these included changes in growth-factor dependence, cell-cycle kinetics, cell adhesion, and gene expression. The investigators concluded that genetic or epigenetic abnormalities acquired during *in vitro* culturing of the NSCs may have accounted for the neural-to-blood fate switch originally observed. Two other recently published articles offer further insights into the possible genetic changes that could confer transdifferentiation potential to otherwise lineage-restricted HSCs and NSCs[117,118]. Both groups set out to examine whether extracellular cues provided by a heterologous microenvironment could alter cell fate decisions of somatic stem cells by co-culturing them with embryonic stem cells. Each obtained results consistent with apparent dedifferentiation to a pluripotent stem cell-like state. However, further investigation revealed that in both cases “dedifferentiation” occurred through spontaneous generation of hybrid cells and not epigenetic reprogramming of the somatic cell genome due to extrinsic signals. Collectively, these new findings underscore the necessity for critical evaluation of all future data from somatic stem cell plasticity experiments. While there is no question that reprogramming of somatic cell nuclei can occur[119,120], the new research suggests that rare transformation events and cell fusion with host cells need to be excluded to convincingly demonstrate that transdifferentiation of somatic stem cells across conventional lineage boundaries has indeed taken place.
